# The Role of Plant-Derived Natural Products in the Management of Inflammatory Bowel Disease—What Is the Clinical Evidence So Far?

**DOI:** 10.3390/life13081703

**Published:** 2023-08-08

**Authors:** Mariela Martinez Davila, Efstathia Papada

**Affiliations:** Division of Medicine, University College London, London WC1E 6JF, UK

**Keywords:** phytochemicals, inflammatory bowel disease, gut microbiota, Mastiha, Curcumin, *Boswellia serrata*, *Artemisia absinthium*

## Abstract

Inflammatory bowel diseases (IBD), including Crohn’s disease (CD) and ulcerative colitis (UC), are a major healthcare challenge worldwide. Disturbances in the immune system and gut microbiota followed by environmental triggers are thought to be part of the aetiological factors. Current treatment for IBD includes corticosteroids, immunosuppressants, and other biologic agents; however, some patients are still unresponsive, and these are also linked to high financial load and severe side effects. Plant-derived natural products are rich in phytochemicals and have been used as healing agents in several diseases since antiquity due to their antioxidant, anti-inflammatory, and immunomodulatory properties, as well as gut microbiota modulation. Numerous in vitro and in vivo studies have shown that phytochemicals act in key pathways that are associated with the pathogenesis of IBD. It is also reported that the use of plant-derived natural products as complementary treatments is increasing amongst patients with IBD to avoid the side effects accompanying standard medical treatment. This review summarises the relevant evidence around the use of plant-derived natural products in the management of IBD, with specific focus on the clinical evidence so far for Curcumin, Mastiha, *Boswellia serrata,* and *Artemisia absinthium*.

## 1. Introduction

Inflammatory bowel disease (IBD) is a gastrointestinal illness that is characterised by chronic inflammation of the gastrointestinal (GI) tract, with the main two types including ulcerative colitis (UC) and Crohn’s disease (CD) [1]. UC and CD are characterised by periods of relapse and remission, and despite sharing similar characteristics and symptoms such as abdominal pain, fatigue, reduced appetite, and diarrhoea, [2], they differ in several aspects. UC is characterised by a continuous pattern of inflammation that can affect the colon and rectum at the level of the mucosa [3], while CD can attack any part of the GI tract (mouth to anus) with transmural inflammation in an intermittent pattern [4].

Over the last few decades, a global rise in the incidence of IBD has been observed, and although IBD has been traditionally considered a disease of the Western world, there is an increasing incidence in newly industrialised countries in Asia, including China and India, as emerging data have shown. However, the reasons for this swift increase in the occurrence of IBD are not fully understood [5].

The pathogenesis of IBD has not been fully elucidated yet, although it seems to be a result of an abnormal response to normal antigens of the gastrointestinal tract. More specifically, a combination of genetic, immunological, and environmental factors initially contributes to destruction of the intestinal epithelial barrier and an increase in intestinal permeability, leading to an influx of immune cells in the intestinal lumen. This dysregulation of the immunomodulation of intestinal mucosa leads to abnormal function of activated T cells, mononuclear cells, and macrophages causing lesions in epithelial cells and chronic inflammation [6]. Perturbations in gut microbiota characterised by depleted diversity, reduced abundance of short chain fatty acid (SCFA) producers, and enriched proinflammatory microbes such as adherent/invasive *Escherichia coli (E. coli)* and H2S producers [7], may also contribute to the inflammatory state in IBD through affecting either the immune system or the metabolic pathways.

The medical management of CD and UC includes a wide range of pharmacological agents such as aminosalicylates, corticosteroids, immunomodulators, biological therapy, and antibiotics. Surgical management is considered when patients do not respond to the medications, and it is still necessary in 30–40% of patients with CD and 20–30% of patients with UC [8]. The main goal of the conventional treatment is to induce and maintain disease remission by achieving clinical and endoscopic healing. Unfortunately, some patients do not respond to the treatment, and usually, several medications are associated with serious side effects. Also considering the chronic, progressive nature of IBD and the increased economic burden of the medical treatment, dietary interventions and plant-derived natural products are gaining attention as alternative or complementary therapies as some patients seek a more natural approach in long-term IBD management [9].

Plant-derived natural products have been well known for their favourable effects on human health since antiquity for pain management, wound healing, gut health, and several other indications. As such, they have attracted scientific interest over the last decades with several studies on identification, isolation, and quantification of their compounds and their biomedical properties. Most of the evidence regarding the effects on plant-derived natural products on IBD comes from in vitro studies and animal models, but there are also numerous clinical trials which have been conducted in humans.

Within this narrative review, we will recap the main mechanisms of action of phytochemicals on IBD, as well as the current evidence from clinical trials investigating the effects of selected plant-derived bioactive compounds rich in phytochemicals on the clinical course of IBD with a specific focus on their anti-inflammatory, antioxidant, and immunomodulatory effects, as well as their impact on gut microbiota modulation. There are several plant-derived natural products evaluated in vitro and in animal models of IBD, but within this review, we focused on reviewing the evidence on some of the most well studied products in human trials in IBD, including curcumin, Mastiha, *Boswellia serrata (BS),* and *Artemisia absinthium (AA)*. The primary search tools in this literature review were Pubmed and Google Scholar. We used keywords and search terms such as “phytochemicals and IBD”, “phytochemicals and UC”, “phytochemicals and CD”, “natural products and IBD”, “natural products and UC”, “natural products and CD”. Human trials that evaluated the therapeutic effects of natural products in IBD were included. Additional searches were performed using “Curcumin”, “Mastiha”, “Mastic gum”, “Chios mastic”, “*Pistacia lentiscus*”, “*Boswellia serrata*”, and “*Artemisia absinthium*”. The reference lists from the retrieved articles were also checked for other relevant studies.

## 2. Plant-Derived Natural Products in IBD

Plant-derived natural products have been traditionally used to provide benefits in terms of health and disease. Their favourable effects have been attributed mainly to their content in phytochemicals of bioactive elements that are naturally present in fruits, vegetables, grains, and other plants [10], and that exhibit antioxidant, anti-inflammatory, and anti-proliferative activity [11]. There are around 10,000 different phytochemicals, and they are categorised mainly into polyphenols, glycosinolates, carotenoids, alkaloids, and terpenes [12]. In vitro and animal models have shown that these are implicated in several biological processes, including scavenging of free radicals, induction of anti-inflammatory responses, maintenance of the homeostatic regulation of the gut microbiota, activation of the intestinal T regulatory cells, maintenance of the mucosal barrier integrity, and inflammatory pathway control [13].

**Figure 1:**
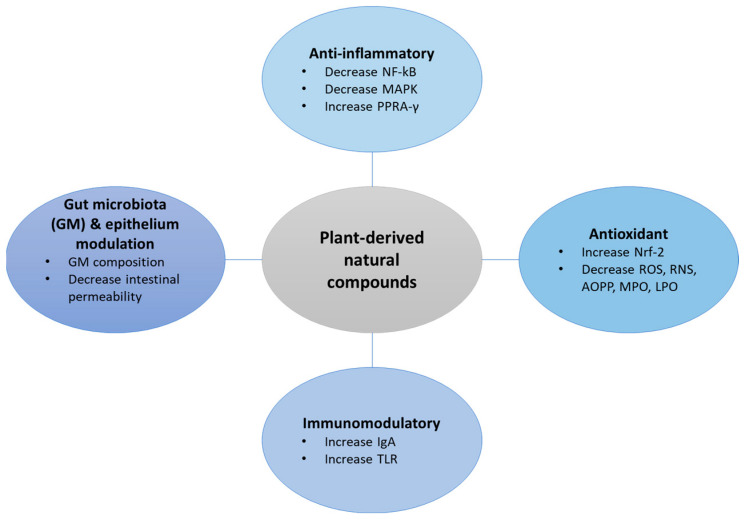
Effects of plant-derived natural compounds on IBD. AOPP = advanced oxidation protein products, IgA = immunoglobulin A, LOP = lipid-oxidised products, NF-kB = nuclear factor kappa B, MAPK = mitogen-activated protein kinase, MPO = myeloperoxidase, PPAR-γ = peroxisome proliferator-activated receptor, ROS = reactive oxygen species, RNS = reactive nitrogen species, TNF-a = tumour necrosis factor alpha, TLR = Toll-like receptors, Nrf-2 = nuclear factor erythroid 2–related factor 2.

Phytochemicals have shown to be effective in key pathways that are associated with the pathogenesis of IBD, as shown in Figure 1, especially through cytokine regulation and reduction in oxidative stress [14,15]. Some of the mechanisms implicated in the pathogenesis of IBD are the release of tumour necrosis factor-alpha (TNF-a) from infiltrating immune cells partnered with the overexpression of nuclear factor kappa B (NF-kB); increased cytokine concentrations, such as interleukin (IL)-6, IL-12 in CD, and IL-13 in UC [16]; and increased production of reactive oxygen species (ROS) exacerbating oxidative stress [17]. These factors create a vicious cycle that eventually leads to the disruption of gut homeostasis and altered intestinal function [18].

Additionally, there is strong evidence that the pathogenesis of IBD is also related to toll-like receptors (TLRs), important mediators of inflammatory pathways in the gut, which play a key role in mediating immune responses. TLR4, one class of TLRs, is thought to play a role in intestinal inflammatory diseases, as it is implicated in the maturation of dendritic cells and differentiation of T helper cells (Th1 and Th2). Furthermore, it can induce the differentiation of macrophages to an M1 phenotype, therefore producing proinflammatory cytokines [19]. TLR4 can also trigger the activation of NF-kB and mitogen-activated protein kinases (MAPKs), as well as the induction of cytokines and inflammation-related enzymes [20]. Studies suggest that certain phytochemicals, such as polyphenols, exert their anti-inflammatory effects through the inhibition of TLR4/NF-kB-mediated signalling pathways and by downregulating the expression of pro-inflammatory mediators [19].

Phytochemicals’ health benefits are also linked with the regulation of different microRNAs, which are implicated in the regulation of the intestinal epithelial barrier, T-cell differentiation, and the Th17 signalling pathway. They also interfere in some of the inflammatory signalling pathways (NF-kB) and signal transducer and activator of transcription STAT/IL-6 pathways [21].

## 3. Modulation of Gut Microbiota

The gut microbiota (GM) can be defined as the community of microorganisms in the gastrointestinal tract and seems to play an important role in human health, including in terms of immune, metabolic and neurobehavioural traits [22]. IBD has been associated with dysbiosis, which is defined as a decrease in gut microbial diversity due to an imbalance between commensal and pathogenic microorganisms [23]. The GM of patients with IBD is characterised by low microbial diversity and decreased abundance of *Bifidobacterium* spp., *Lactobacillus* spp., *and F. prausnitzii,* while having an abundance of pathologic bacteria such as *E. coli* and *Clostridium difficile (C. difficile)* [7].

Phytochemicals in plant-derived natural products are poorly absorbed in the small intestine; therefore, they reach the large intestine, where they can exert a prebiotic and selective antimicrobial effect [24]. The proposed mechanism behind these health benefits is through the metabolites produced by the GM, rather than the bioactive compounds per se [25]. These metabolites can effectively modulate the GM mainly by inducing the growth of some beneficial bacterial populations, such as *Lactobacillus* spp. and *Bifidobacterium* spp., and by triggering the generation of SCFAs [25]. One example is resveratrol, a polyphenol found in berries, grapes, and groundnuts, which showed an increase in the abundance of *Lactobacillus* spp. and *Bifidobacterium* spp. while inhibiting the production of harmful bacteria such as *E. coli* in mice models [26,27]. Additionally, another trial showed that mice fed with resveratrol had an increased amount of SCFA producers like *Butyricicoccus* spp., *Ruminococcus* spp., and *Roseburia* spp. [28]. Furthermore, epigallocatechin (EGCG), a polyphenol found in green tea, showed a prebiotic effect by significantly enriching SCFA-producing bacteria, such as *Akkermansia* spp., while attenuating colitis symptoms in a mouse model of UC when supplemented as prophylaxis [29].

Other mechanisms by which phytochemicals and GM are related include the interaction with the immune system, especially by immunoglobulin A (IgA), regulating the degree of colonisation and preventing dysbiosis [30], as it has been reported with some carotenoids (b-carotene and astaxanthin) [31]. They also include influence on the mucin layer and tight junction integrity [32], as well as synergistic anti-inflammatory effects with other compounds like omega-3 fatty acids [33].

## 4. Evidence from Human Studies

As mentioned above, plenty of in vitro and animal studies have evaluated the effects of plant-derived natural products in IBD. Data from human studies are also available, but to a significantly lesser extent compared to animal and in vitro studies. However, as it is estimated that around 21–60% of patients with IBD use complementary or alternative therapies (herbal remedies, dietary supplements) due to a perception of less toxicity or harm, and up to 75% do not discuss it with their physicians [34], it is essential to explore the current evidence regarding the benefits and risks. In this part we will discuss the clinical evidence on the effects of some plant-derived natural products, including curcumin, Mastiha, *Boswellia serrata,* and *Artemisia absinthium* on patients living with IBD.

### 4.1. Curcumin

**Figure 2:**
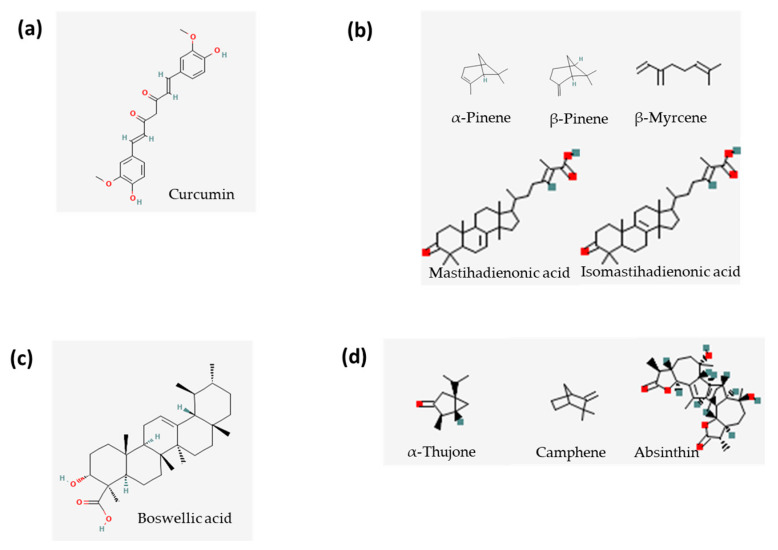
Main bioactive compounds of (**a**) *Curcuma longa* (curcumin [38]); (**b**) Mastiha (α-pinene [39], β-pinene [40], β-myrcene [41], mastihadienonic acid [42], isomastihadienonic acid [43]); (**c**) Boswellia serrata (boswellic acid [44]); (**d**) Artemisia absinthium (α-thujone [45], camphene [46], absinthin [47]). Source of chemical structures: PubChem [48].

Curcumin is the biologically active, phenolic component of turmeric (*Curcuma longa*) [35]. Turmeric consists of several significant constituents isolated from the rhizome—structurally-related curcuminoids, including curcumin as the most important and the main active compound (Figure 2a). It has been characterised by antimicrobial, antioxidant, and immunomodulatory properties in several in vitro and in vivo studies, and it is one of the most evaluated compounds in humans. In the intestinal mucosa, curcumin can reduce the levels of ROS, superoxide anions, and malondialdehyde (MDA) [36]. Additionally, curcumin strongly inhibits the expression of NF-kB and downstream signalling of proinflammatory cytokines such as TNF-a [37].

Several trials in patients with UC showed positive results in terms of inducing remission with no adverse effects, as well as improving endoscopic and clinical activity (Table 1). A pilot study in five patients with UC reported a significant improvement in the number and quality of stools (*p* < 0.02) with the supplementation of 550 mg of curcumin twice a day for one month and then 550 mg three times a day for another month, as assessed by a global score. In the same study, curcumin’s efficacy as an add-on therapy to existing treatments of CD was also tested using 360 mg of curcumin three times a day for one month, and then four times a day for two months, in five patients with CD. The Crohn’s Disease Activity Index (CDAI) score and CRP levels for all subjects fell [49]. Another trial compared curcumin supplementation combined with either sulfasalazine or mesalamine versus placebo in eighty-nine patients with quiescent UC. Patients received curcumin capsules (2 g/day) for six months. The results showed an improvement both in the clinical activity index (CAI) and the endoscopic index (EI), therefore suppressing the morbidity associated with UC [50].

Given the high use of complementary alternatives, a forced-dose titration study in patients with CD and UC tested curcumin’s tolerability and safety. Patients initially received 500 mg twice per day for three weeks, and the dose was increased up to 1 g twice per day at week 3 for a total of 3 weeks, and then titrated again to 2 g twice per day at week 6 for three weeks. Validated measures of disease activity were used, such as the Paediatric Ulcerative Colitis Activity Index (PUCAI), Paediatric Crohn’s Disease Activity Index (PCDAI), and Monitoring of Side Effect System Score, and all patients tolerated curcumin well with no serious side effects reported [51]. It is essential to mention that this study was performed in a paediatric population; therefore, further research is required in order to fully assess the safety and efficacy in larger studies including patients of different ages.

Forty-five patients with distal UC were randomised to either NCB-02 enema (140 mg of NCB-02 (curcumin) preparation dissolved in 20 mL of water) or placebo, both complemented with oral 5-ASA (800 mg twice daily), for eight weeks. At week 8, clinical remission and improvements in endoscopy were higher in the NCB-02 group compared to the placebo, but the difference did not reach statistical significance [52].

Curcumin’s efficacy was studied as an add-on therapy with mesalamine treatment (3 g/day for 1 month with continued mesalamine) to induce remission in fifty patients with active UC. The combination of curcumin and mesalamine was significantly superior to the combination of placebo and mesalamine in clinical (reduction > 3 points in Simple Clinical Colitis Activity Index (SCCAI)) and endoscopic remission (partial Mayo score < 1) [53]. Disease activity improvement was reported in seventy patients with mild-to-moderate UC when supplemented with curcumin 1500 mg/day for 8 weeks compared to placebo [54]. Clinical outcomes and quality of life were significantly improved in curcumin-treated patients. Additionally, curcumin supplementation reduced serum high-sensitivity C-reactive protein (hs-CRP) concentration and the erythrocyte sedimentation rate (ESR) significantly, biomarkers which are usually elevated in IBD.

The increased interest in using supplements as a co-adjuvant treatment for IBD has led to the development of products with enhanced properties; new forms of curcumin have been synthesised with higher absorption rates than conventional curcumin powder [55]. A nanomicellar curcuminoid formula with higher bioavailability than conventional formulas was used to test the effectiveness of the treatment of UC. Fifty-six patients with mild-to-moderate UC were randomly assigned to receive the formula (80 mg, 3 times/day orally) plus 3 g/day mesalamine or placebo plus mesalamine for 4 weeks [56]. There was a significant improvement in the score for urgency of defecation in the formula supplemented group, while an increase in self-reported well-being and reduced clinical activity were also reported.

Another study in thirty patients with CD tested a newly synthesised curcumin derivative (Theracurmin) with a higher absorption rate for 12 weeks, showing a significant improvement in clinical remission rates (*p* = 0.020) and endoscopic measures (*p* = 0.032) compared with the placebo with no adverse effects [57]. Lastly, another bioenhanced form of curcumin with higher bioavailability (BEC) was studied versus a placebo in sixty-nine patients with mild-to-moderate UC on standard doses of mesalamine for up to 12 months [58]. Clinical response, clinical remission, and endoscopic remission were evaluated at 6 weeks and 3 months, then followed up at 6 and 12 months to assess the maintenance of remission. At 6 weeks, these were significantly higher in the BEC group compared with the placebo. A total of 95% of BEC responders maintained clinical remission compared to none in placebo group at 6 months [58].

Although the aforementioned studies have reported some promising results regarding the use of curcumin as an adjunct to the standard medical treatment for patients with IBD, a prospective randomised double-blind placebo-controlled trial comparing the remission-inducing effect of oral curcumin and mesalamine using a placebo or 2.4 g of mesalamine in patients with ulcerative colitis of mild-to-moderate severity concluded that a dose of 450 mg/day for 8 weeks was not effective in terms of inducing remission [59]. Another study in patients with CD undergoing bowel resection was performed to assess whether curcumin was efficient for the prevention of post-operative recurrence of CD [60]. Patients received 3 g/day oral curcumin plus azathioprine 2.5 mg/day or an identical placebo for 6 months. The results suggested that curcumin was no more effective than the placebo in preventing CD recurrence, as no significant differences between groups were reported. Thus, there is still need for further research with increased sample sizes to be able to reach safe conclusions on the use of curcumin in the management of IBD.

**Table 1:** Clinical evidence on the effects of curcumin on IBD.

Aspect Evaluated	Sample	Duration	Dose	MAIN Outcomes	Reference
Effect on IBD	5 patients with UC	2 months	550 mg of curcumin twice daily for 1 month and then 3 times/d for another month for UC	Improved global score (number and quality of stools) (*p* < 0.02), serologic indexes, sedimentation rate, and CRP decrease within normal limits	[49]
	5 patients with CD	3 months	360 mg of curcumin 3 times/day for 1 month and then 360 mg 4 times/day for 2 months	Decrease in CDAI (mean reduction of 55 points); decrease in sedimentation rate (mean reduction of 10 mm/h); CRP was reduced by a mean of 0.1 mg/dL	
Efficacy as a maintenance therapy in quiescent UC	89 patients with quiescent UC	6 months	Curcumin capsules 2 g/day plus sulfasalazine or mesalamine or placebo plus sulfasalazine or mesalamine	Improved clinical activity index (CAI) (*p* = 0.038) and endoscopic index (EI) (*p* = 0.0001)	[50]
Tolerability in children with IBD	11 patients with IBD(6 with CD and 5 UC)	9 weeks	500 mg curcumin twice daily plus 1 g increase twice daily at week 3 plus 2 g twice daily at week 6	Curcumin was well tolerated in doses up to 2 g twice per day	[51]
Efficacy and safety in distal UC	45 patients with distal UC	8 weeks	Curcumin enema plus oral 5-ASA or placebo enema plus 5-ASA	Higher remission in patients with curcumin treatment (*p* = 0.14); improvement in endoscopic parameters (*p* = 0.29)	[52]
Add-on therapy with optimised mesalamine in UC	50 patients with active mild-to-moderate UC	1 month	3 g/day curcumin capsules with continued mesalamine or placebo capsules plus mesalamine	Higher clinical improvement and remission in patients on curcumin vs. placebo (*p* < 0.01) Higher endoscopic remission in the curcumin group vs. placebo (*p* = 0.043).	[53]
Effect on inducing remission in UC	62 patients with active mild-to-moderate UC	8 weeks	450 mg/day curcumin plus mesalamine OR placebo plus mesalamine	No significant differences between groups	[59]
Effect of a nano formulation of curcuminoids in UC	56 patients with mild-to-moderate UC	4 weeks	80 mg curcuminoid formula, 3 times/day orally plus mesalamine or placebo plus mesalamine	Score for urgency of defecation reduced; mean SCCAI score lower in curcuminoid group (*p* = 0.050)	[56]
Theracurmin efficacy and safety in CD.	30 patients with active-to-moderate CD	12 weeks	360 mg/day Theracurmin or placebo	CDAI score significantly decreased (*p* = 0.005) in Theracurcumin group Reduction in endoscopic CD severity (*p* = 0.032) compared to baseline	[57]
Effect on improvement of disease activity in UC.	70 patients with mild-to-moderate UC	8 weeks	1500 mg/day curcumin or placebo	Improvement in clinical outcomes Changes in SCCAI (*p* < 0.001) and IBDQ (*p* = 0.006) higher in curcumin group; serum hs-CRP and ESR decreased (*p* = 0.01 and *p* = 0.02)	[54]
Effect in preventing post-operative recurrence of CD	62 patients with CD undergoing bowel resection	6 months	Azathioprine (2.5 mg/kg) plus oral curcumin (3 g/day) or placebo	Curcumin not more effective than placebo to prevent CD recurrence.	[60]
Efficacy of a novel bio enhanced curcumin as add on therapy in UC.	69 patients with mild-to-moderate UC	Up to 12 months (6-week, 3-month, 6-month, and 12-month follow-ups)	50 mg of bio-enhanced curcumin (BEC) twice a day or placebo plus standard dose of mesalamine	Higher clinical and endoscopic remission compared to placebo (*p* < 0.01) at 6 weeks Maintenance of remission in 95% (6-mo) and 84% (12-mo) of BEC responders compared to none in the placebo.	[58]

IBD = inflammatory bowel disease, UC = ulcerative colitis, CRP = C-reactive protein, CDAI = Crohn’s Disease Activity Index, CAI = Clinical Activity Index, EI = Endoscopic Index, CD = Crohn’s Disease, SCCAI = Simple Clinical Colitis Activity Index, IBDQ = Inflammatory Bowel Disease Questionnaire, ESR = erythrocyte sedimentation rate, hs-CRP = high-sensitivity C-Reactive Protein, BEC = bio-enhanced curcumin.

### 4.2. Mastiha

Mastiha, is a natural product of the Mediterranean basin consisting of a plethora of bioactive constituents, including phenolic compounds, phytosterols, arabino-galactanes proteins, and 30% of a natural polymer (poly-β-myrcene). However, it is a particularly concentrated source of terpenes, such as monoterpenes (i.e., α-pinene, β-pinene, and β-myrcene) and triterpenes (i.e., mastihadienonic acid and isomastihadienonic acid) (Figure 2b) [61]. It is obtained as a dried resinous exudate from stems and branches of the tree *Pistacia lentiscus (Pistacia lentiscus* L*. var latifolius Coss or Pistacia lentiscus var. Chia*) and has been used since antiquity for its anti-inflammatory and antioxidant properties [62]. The European Medicines Agency has recognised Mastiha as a herbal medicinal product for the following indications: (a) mild dyspeptic disorders and (b) symptomatic treatment of minor inflammations of the skin and as an aid in the healing of minor wounds [63]. Previous data on Mastiha has suggested that it could have favourable effects on the clinical course and inflammatory biomarkers of patients with IBD (Table 2). A pilot study in patients with active CD evaluated the effectiveness of Mastiha administration on the clinical course and blood inflammatory markers. The study included a healthy control group, and participants received 4 weeks of treatment with Mastiha capsules (6 capsules/day, 0.37 g/cap). The results suggested that Mastiha significantly decreased the CDAI and plasma levels of IL-6 and CRP [64]. Peripheral blood mononuclear cells (PBMC) were also evaluated before and after treatment, showing a reduction in TNF-alpha secretion and an increase in macrophage migration inhibitory factor (MIF) release. These findings pointed towards an inhibition of random migration and chemotaxis of monocytes/macrophages [65]. A randomised, double-blind, placebo-controlled trial in patients with IBD assessed the effects of a Mastiha supplement on oxidative stress biomarkers and the plasma-free amino acid (AA) profiles of patients with active IBD (CD and UC). Participants were allocated to receive Mastiha (2.8 g/day) or a placebo for three months, being either under no treatment or under stable medical therapy. A favourable effect on oxidative stress biomarkers was documented, as oxidised low-density lipoprotein (OxLDL), OxLDL/HDL, and OxLDL/LDL decreased significantly in the Mastiha group. Mastiha also ameliorated a reduction in plasma free AAs in patients with UC taking the placebo [66]. It has been proposed that Mastiha can also exert a prebiotic effect, as it showed a regulatory role in faecal lysozyme. In addition to a significant improvement in the IBDQ score, results from the same trial showed a significant decrease in faecal lysozyme in the Mastiha group [67]. Lysozyme is an antimicrobial protein that regulates innate immune response, and its increased expression is correlated with dysbiosis and inflammation [68], suggesting that Mastiha’s effect could be prebiotic as well.

A randomised, double-blind, placebo-controlled clinical trial in 68 patients with IBD in remission examined the effects of Mastiha on the clinical course and AA profiles of patients. The results showed that AAs such as valine, alanine, proline, glutamine, and tyrosine significantly increased only in the placebo group compared with the baseline, and the change between the two groups was significantly different. As AAs are considered an early prognostic marker of disease activity, these results may suggest a potential role of Mastiha in remission maintenance, although Mastiha was not proven superior to the placebo in terms of the remission rate [69].

In recent years, biological therapies that target different molecular pathways have been developed, such as TNF blockade, IL-6, IL-12/IL-23, and IL-17 pathways. IL-17 blocking agents have been applied in several anti-inflammatory diseases, but unfortunately, in IBD, clinical benefits have not been established without adverse effects. Mastiha has been shown to act in the IL-17 pathway, and its use as a safe adjunct therapy has been proposed in IBD. A double-blind, placebo-controlled, parallel group study explored Mastiha’s immunomodulatory effect on IL-17A serum levels in patients with active and inactive IBD. IL-17A has been studied for its anti-inflammatory nature and a potential protective role in intestinal pathology. The participants received natural Mastiha at a dose of 2.8 g/day or an identical placebo (6 months for patients in remission and 3 months for patients in relapse). The results showed a significant increase in serum IL-17A in patients with inactive UC, while decreasing significantly only with the placebo treatment. No significant differences were reported in active disease. Additionally, Mastiha seemed to influence the stool metabolic profile of patients in remission, as there were increased levels of glycine and tryptophan, both related to therapeutic effects and immunoregulatory mechanisms, such as Th17 cell differentiation. These findings suggest that Mastiha has a potential immunomodulatory role in quiescent IBD [70].

MicroRNAs (miRNAs), which are one of the most studied epigenetic mechanisms, are implicated in the regulation of the intestinal barrier and cell membrane trafficking. They also interfere with inflammatory pathways, such as NF-kB and the signal transducer and activator of the transcription (STAT)/IL-6 pathways. In IBD, miRNAs are usually up-regulated and are also involved in T-cell differentiation and Th17 signalling pathways (especially microRNA-155). Circulating miRNAs are considered a valuable tool to indicate the physiological state of the tissue from which they come, and their modulation by certain phytochemicals has been studied over the years. A study in subsets of patients with inflammatory conditions, including IBD, evaluated whether a common route exists in the anti-inflammatory activity of Mastiha, specifically through the regulation of miRNA levels. Participants received Mastiha in tablets at doses of 2.8 g/day or identical placebo tablets adjunct to conventional medical treatment (6 months for patients in remission and 3 months for patients in relapse). The results showed that, particularly in patients with UC in relapse, miRNA-155 increased in the placebo group significantly, while this increase was prevented by mMastiha supplementation. These findings proposed a regulatory role for Mastiha in circulating levels of miR-155, a critical player in Th17 differentiation and function [21]. However, this was the first study linking Mastiha to epigenetic mechanisms; therefore, it should be confirmed by more studies in larger cohorts.

### 4.3. Boswellia serrata

*Boswellia serrata (BS)* is a gum resin rich in terpenes, such as boswellic acid (Figure 2c). It is obtained from the *Boswellia serrata* tree, but evidence regarding its effects on human IBD has been contradictory so far (Table 3). There are several mechanisms of action that contribute to its anti-inflammatory and immunomodulatory activities, such as the ability to inhibit the formation of leukotrienes, which act as potent mediators of inflammatory disorders [71]. A randomised, double-blind, verum-controlled, parallel group study in 102 patients with active CD showed that a BS extract could be as effective as mesalamine, but not superior, in reducing the CDAI score [72]. However, a double-blind, placebo-controlled, randomised, parallel study in 108 outpatients with CD in clinical remission randomised to Boswelan, a BS extract, (3 × 2 capsules/day; 400 mg each) or placebo for 52 weeks was unable to demonstrate superiority in maintaining remission when compared to the placebo, although it showed good tolerability [73]. As bioavailability has been a topic of concern, a novel delivery form of *Boswellia serrata* extract (BSE) with enhanced bioavailability was tested [74]. The study was conducted in 43 patients with UC supplemented with either 250 mg/day of BSE or no supplement for 4 weeks. The results showed a significant positive effect in the supplemented group for all the evaluated parameters, such as intestinal pain, evident and occult blood in stools, bowel movements and cramps, watery stools, etc. The faecal concentration of calprotectin was also significantly decreased in the supplemented group [74]. The present results highlight the importance of future research that will assess the effects of BS, both in maintaining remission and in the management of active disease.

### 4.4. Artemisia absinthium

Also known as wormwood, *Artemisia absinthium (AA)* is a herbaceous plant considered a very important species in the history of medicine [75]. It contains several groups of phytochemicals (Figure 2d), such as terpenes (e.g., α-thujone, camphene), lactones (e.g., absinthin), flavonoids, phenolic acids, coumarins, and tannins; therefore, it has been widely used as a therapeutic aid in digestive disorders and other clinical conditions [75]. Some in vitro studies have reported that Artemisia species and isolated compounds mainly act by supressing TNF-a and other interleukins [76,77].

A double-blind placebo study in CD patients assessed whether *AA* could reduce the patients’ dependence on corticosteroids (Table 4). Participants receiving a daily dose of steroids at an equivalent of 40 mg or less of prednisone for at least three weeks were administered an *AA* containing herbal blend capsule (3 × 500 mg/day) or placebo for ten weeks. The steroid dose was maintained stable until week two; after that, tapering off started and was completed at week 10. Already, after six weeks, there was a significantly higher number of patients who showed clinical improvement (CDAI score of 70 or more) in the *AA* group as compared to placebo, and this continued beyond week 10. Improvements were also reported in mood and quality of life, as measured with the 12-item Hamilton Depression Scale (HAMD). These findings suggest that *AA* might have a steroid-sparing effect on CD patients. However, it is important to mention that five patients from this group showed little response to the *AA* treatment, suggesting that there could be a group of patients who are resistant to the treatment [78].

As mentioned previously, TNF-a appears to play a central role in the pathogenesis of CD. A clinical trial studied whether the *AA* TNF-a suppression effect was present in 20 patients with CD. Patients were given, in addition to their basic CD therapy, a dried powdered *AA* treatment (3 × 750 mg) for 6 weeks. A control group was included. The results showed a significant decrease in TNF-a serum levels in the *AA* group as compared to the control group. Additionally, *AA*-treated patients showed significant clinical improvements in their CDAI, IBDQ, and HAMD scores. These findings suggest that *AA* can be a potential adjuvant for the TNF-a-mediated diseases, although further research is needed [79]. 

## 5. Bioavailability and Safety Considerations

Bioavailability can be defined as the fraction of the active form of a substance that reaches systemic circulation unaltered and is absorbed and used by the body [80]. Some phytochemicals show low levels of stability, as they are highly metabolised or rapidly eliminated [81]; this has been a challenge when using them as therapeutic agents [82], and the research in this field is of great importance for their clinical use.

Many plant-derived products show remarkable potential in vitro, but due to their poor absorption, the effect in vivo has not been completely demonstrated [83]. Several factors, such as the transformation during metabolic pathways or during processing [84], solubility [83], gender differences, and dosage seem to affect bioavailability [85,86]. Previous research on phytochemical bioavailability showed a peak in plasma antioxidant capacity 1–2 h after intake [87]; however, research on the absorption, metabolism, and distribution of these compounds in the human body remains limited. Different strategies can be implemented to increase bioavailability, such as extraction of the active ingredient or combination with other compounds that enhance absorption rates and could be of great therapeutic use. For example, evidence shows that the absorption of curcumin is increased by 20 times in humans and 1.56 times in rats when co-administered with 20 mg of piperine [88]. However, a recent study reported that liver injury due to turmeric appears to be increasing in the United States, perhaps reflecting usage patterns or increased combination with black pepper [89], indicating that further research regarding safety and education of the public on the use of plant-derived natural compounds is necessary. Data on safety are very limited for the majority of these compounds, since most studies are short-term and use different formulations and dosages, making it difficult to reach safe conclusions.

## 6. Future Implications and Conclusive Remarks

IBD incidence has been rising exponentially worldwide, and an attempt to improve treatment has been a priority over the years. The recurrent pattern of IBD demands constant treatment, representing a significant financial load to individual patients and healthcare systems, especially in developing countries. Additionally, there are still many challenges, as many adverse effects and/or patients who are non-responsive to treatments are still reported. Emerging evidence shows that a combined treatment using standard medications and plant-derived natural products might provide higher remission rates and decrease adverse effects in IBD. The use of plant-derived natural products in patients with IBD has demonstrated improvement in several aspects assessed by tools such as CDAI, IBDQ, and SCAAI scores, which have been validated and implemented in clinical practice. However, it is worth mentioning that these improvements might not always correlate with endoscopic scores or faecal biomarkers of inflammation, and the indices currently in use are limited to predicting long-term outcomes like surgery, relapse, and disability. Even though evidence on this topic has been increasing, human trials are very limited and sample sizes are quite small. Therefore, it is essential to prioritise clinical trials in order to facilitate the development of official recommendations or guidelines on how to use them safely. Further research focusing on phytochemical bioavailability, optimal doses, and safety is needed for the development of phytochemical-rich products with enhanced therapeutic properties for chronic inflammatory conditions, including IBD.

## Figures and Tables

**Table 2:** Clinical evidence on the effects of Mastiha on IBD.

Aspect Evaluated	Sample	Duration	Dose	Main Outcomes	Reference
Effects on the clinical course of active CD	10 patients with active CD and 8 healthy controls	4 weeks	6 Mastiha caps/day (2.2 g/day)	Significant reduction in CDAI (*p* = 0.05), CRP (*p* = 0.028), and plasma IL-6 (0.027). Total antioxidant potential (TAP) significantly increased in the Mastiha group (*p* = 0.036)	[64]
Effects on cytokine production of circulating mononuclear cells in active CD				Reduction in TNF-a secretion from PBMC in the Mastiha group (*p* = 0.028) and an increase in MIF (*p* = 0.026). No significant changes in IL-6, MCP-1, or GSH.	[65]
Effects on oxidative stress and plasma-free amino acid profile in active IBD	60 patients with active IBD	3 months	2.8 g/day Mastiha (4 tabs 700 mg) plus conventional medical treatment or Placebo plus conventional medical treatment	Significant decrease in oxLDL (*p* = 0.031), oxLDL/HDL (*p* = 0.020) and oxLDL/LDL (*p* = 0.005) in the Mastiha group and amelioration of the decrease in plasma-free AAs in patients with UC.	[66]
Effects on QoL, inflammatory biomarkers, and clinical course in IBD				IBDQ significantly improved (*p* = 0.004); significant decrease in faecal lysozymes (*p* = 0.018) and fibrinogen (*p* = 0.006) in the Mastiha group. Significant increase in faecal lactoferrin (*p* = 0.001) and calprotectin (*p* = 0.029) in the placebo group	[67]
Effect on the clinical course and amino acid profile in inactive IBD	68 patients with inactive IBD	6 months	2.8 g/day Mastiha plus conventional medical treatment or Placebo plus conventional medical treatment	Attenuation of the increase in free AA levels in the Mastiha group; significant decrease in oxidative stress biomarkers	[69]
Regulatory effect on IL-17A serum levels in IBD	43 patients with UC and 86 patients with CD	3 months in active and 6 months in inactive IBD	2.8 g/day Mastiha plus conventional medical treatment or Placebo plus conventional medical treatment	Increase in serum IL-17A in the Mastiha group (*p* = 0.006) and significant difference between Mastiha and placebo in the mean change in inactive IBD.	[70]
Anti-inflammatory activity through regulation of miRNA in IBD	60 patients with IBD (endoscopy-proven CD or UC) with usual medical treatment	3–6 months	2.8 g/day Mastiha or placebo	miR-155 increased in the placebo group in active UC (*p* = 0.054), while it was prevented by Mastiha.	[21]

CD = Crohn’s disease, CDAI = Crohn’s Disease Activity Index, CRP = C-reactive protein, IL-6 = interleukin-6, TAP = total antioxidant capacity, TNF-a = tumour necrosis Factor-alpha, PBMC = peripheral blood mononuclear cells, MIF = macrophage migration inhibitory factor, MCP-1 = monocyte chemoattractant protein-1, GSH = glutathione, IBD = inflammatory bowel disease, UC = ulcerative colitis, oxLDL = oxidised LDL, AAs = amino acids, QoL = quality of life, IBDQ = Inflammatory Bowel Disease Questionnaire, IL-17A = interleukin 17A, miRNA = microRNA, miR-155 = microRNA 155.

**Table 3:** Clinical evidence on the effects of *Boswellia serrata* on IBD.

Aspect Evaluated	Sample	Duration	Dose	Main Outcomes	Reference
Safety and efficacy of BS extract H15 on active CD	102 participants	8 weeks	3.6 g/day BS extract H15 OR mesalamine	Reduction in CDAI score, but no significant superiority compared to mesalamine (*p* = 0.061)	[72]
Effect and safety of long-term therapy in CD	108 patients with CD in remission	52 weeks	Boswelan 3 × 2 capsules/day (400 mg each) OR placebo	Good tolerability and safety; no superiority versus placebo as maintenance therapy (*p* = 0.85)	[73]
Effect of BS extract (BSE) in UC	43 participants with UC in remission for at least 1 year	4 weeks	250 mg/day BSE in a novel lecithin-based delivery form (Casperome^®^) OR no supplementation	Significant improvement in diffuse intestinal pain, blood in stools, bowel movements and cramps, and reduction in calprotectin levels (*p* < 0.05)	[74]

CD = Crohn’s disease, CDAI = Crohn’s Disease Activity Index, BS = Boswellia Serrata, BSE = Boswellia Serrata extract, UC = ulcerative colitis.

**Table 4:** Clinical evidence on the effects of *Artemisia absinthium* on IBD.

Aspect Evaluated	Sample	Duration	Dose	Main Outcomes	Reference
Steroid-sparing effect on CD	40 participants with CD	20 weeks	*AA* containing herbal blend (3 × 500 mg/day) (SedaCrohn^®^) plus steroids or placebo	Significantly higher clinical improvement using CDAI in SedaCrohn^®^ group (*p* < 0.01)	[78]
TNF-α suppressing effect on CD	20 participants with active CD	6 weeks	3 capsules SedaCrohn^®^ 3 times/day (250 mg of powdered *AA* each one) plus conventional medical treatment or placebo	Significant reduction in CDAI and TNF-a levels compared with placebo	[79]

CD = Crohn’s disease, CDAI = Crohn’s Disease Activity Index, TNF-a = tumour necrosis factor-alpha, *AA= Artemisia absinthium*

## Data Availability

Not applicable.
